# Vegetative and reproductive growth of salt-stressed chickpea are carbon-limited: sucrose infusion at the reproductive stage improves salt tolerance

**DOI:** 10.1093/jxb/erw177

**Published:** 2016-05-02

**Authors:** Hammad A. Khan, Kadambot H.M. Siddique, Timothy D. Colmer

**Affiliations:** 1 School of Plant Biology, Faculty of Science, The University of Western Australia, 35 Stirling Hwy, Crawley WA 6009, Australia; 2 The UWA Institute of Agriculture, The University of Western Australia, 35 Stirling Hwy, Crawley WA 6009, Australia

**Keywords:** Chickpea (*Cicer arietinum* L.), flowering, photosynthate supply and demand, photosynthesis, plant sucrose infusion, podding, salinity stress, seed growth, tissue ions, tissue sugars.

## Abstract

Reproductive processes of chickpea (*Cicer arietinum* L.) are particularly sensitive to salinity. We tested whether limited photoassimilate availability contributes to reproductive failure in salt-stressed chickpea. Rupali, a salt-sensitive genotype, was grown in aerated nutrient solution, either with non-saline (control) or 30mM NaCl treatment. At flowering, stems were either infused with sucrose solution (0.44M), water only or maintained without any infusion, for 75 d. The sucrose and water infusion treatments of non-saline plants had no effect on growth or yield, but photosynthesis declined in response to sucrose infusion. Salt stress reduced photosynthesis, decreased tissue sugars by 22–47%, and vegetative and reproductive growth were severely impaired. Sucrose infusion of salt-treated plants increased total sugars in stems, leaves and developing pods, to levels similar to those of non-saline plants. In salt-stressed plants, sucrose infusion increased dry mass (2.6-fold), pod numbers (3.8-fold), seed numbers (6.5-fold) and seed yield (10.4-fold), yet vegetative growth and reproductive failure were not rescued completely by sucrose infusion. Sucrose infusion partly rescued reproductive failure in chickpea by increasing vegetative growth enabling more flower production and by providing sucrose for pod and seed growth. We conclude that insufficient assimilate availability limits yield in salt-stressed chickpea.

## Highlight

Salinity damage and reduced photosynthesis in leaves impedes vegetative and reproductive growth of chickpea; substantial recovery of plants with sucrose infused into the stem demonstrated carbon limitation in salt-stressed chickpea.

## Introduction

Chickpea (*Cicer arietinum* L.) is commonly grown in semi-arid regions, but soils in these regions can be salt affected (Ryan, 1997) and chickpea is relatively sensitive to soil salinity (Flowers *et al.*, 2010). Salinity impairs vegetative growth in chickpea (Lauter and Munns, 1986; Dua and Sharma, 1997; Khan *et al.*, 2015), but reproductive growth is particularly salt sensitive (Vadez et al., 2007, 2012; Samineni *et al.*, 2011; Turner *et al.*, 2013). Reproductive failure/success, measured as seed mass per plant, is attributed to the effects of salinity on flower number, ovule fertilization, pod development and retention, seed number and seed size (Flowers *et al.*, 2010). In chickpea, reproductive success under salt stress has been associated with the production of more tertiary branches and flowers, more filled pods and, therefore, more seeds (Vadez et al., 2007, 2012; Kotula *et al.*, 2015). An understanding of which factor(s) limit reproductive growth in salinized chickpea would assist in the development of a physiological-based breeding program to produce varieties with improved salt tolerance.

Salt stress impairs reproductive processes/development in plants due to the possible accumulation of toxic ions (Na^+^ and/or Cl^−^) in reproductive tissues, reduced supply of assimilates to reproductive tissues due to decreased leaf area and reduced photosynthesis, water restriction and/or hormonal imbalances (Khatun and Flowers, 1995; Munns and Rawson, 1999; Munns, 2002; Ghanem *et al.*, 2009). In chickpea, potentially toxic levels of Na^+^ and Cl^−^ were found in flowers, pod walls and seeds (Murumkar and Chavan, 1986; Samineni *et al.*, 2011), but Na^+^ and Cl^−^ concentrations in ovules soon after fertilization were relatively low and did not explain differences in the seed yield of two contrasting genotypes (Kotula *et al.*, 2015). Turner *et al.* (2013) reported that salt stress increased pod abortion in sensitive genotypes, but that pollen viability, *in vitro* pollen germination and *in vivo* pollen growth was not affected. Salinity damaged leaf tissues and decreased photosynthesis in chickpea (Khan *et al.*, 2015), so we hypothesize that a reduction in the availability of photoassimilates to reproductive organs may cause reproductive failure in salt-stressed chickpea.

Low availability of photoassimilates has resulted in reproductive failure in several species under salt stress (wheat and barley, Munns and Rawson, 1999; rice, Abdullah *et al.*, 2001; tomato, Ghanem *et al.*, 2009). Reduced grain production in rice has been linked with reduced production of photoassimilates in leaves and their decreased translocation to the reproductive organs (Sultana *et al.*, 1999), but also to high Na^+^ and Cl^−^ in reproductive organs (Khatun and Flowers, 1995; Khatun *et al.*, 1995). For salinized wheat, 80–90% of carbon in grain-filling comes from current photosynthesis, so reduced photosynthesis can limit grain/seed filling (Khan and Abdullah, 2003). More widely, for crops faced with abiotic stress, reduced availability of photoassimilates is a major factor determining flower, fruit and seed abortion (Ruan *et al.*, 2012). Reproductive failure in drought-stressed maize has been associated with reduced photosynthesis and sugar supply to developing ears, as elegantly demonstrated by the provision of sucrose via stem-infusion, which enhanced reproductive growth (Boyle *et al.*, 1991; Zinselmeier *et al.*, 1995a). To test the hypothesis that reproductive growth in salt-stressed chickpea is limited by low availability of photoassimilates, we infused sucrose into stems with the expectation that sucrose provision would improve vegetative and reproductive growth of chickpea in saline conditions.

## Materials and methods

### Plant materials and growth conditions

The experiment was conducted in a naturally-lit phytotron (temperature-controlled glasshouse; 20/15±2 °C day/night) during winter and spring 2013 (August to November) at The University of Western Australia, Perth, Australia (31°57′ S, 115°47′ E). Seeds of a salt-sensitive genotype (Rupali) of desi chickpea (Khan *et al.*, 2015; Kotula *et al.*, 2015) were washed with 0.042% (w/v) sodium hypochlorite for 5min and rinsed twice in tap water. The seed coat was pricked with a needle and seeds were imbibed overnight in an aerated solution of 0.5mM CaSO_4_ in darkness. Imbibed seeds were germinated on mesh in a 10% concentration of the aerated nutrient solution (see below for composition) in the dark for 4 d and then seedlings were transferred to a 25% concentration of aerated nutrient solution exposed to light for another 8 d. Twelve days after imbibition, seedlings were transferred to 4.5 l plastic pots (two per pot) containing full-strength aerated nutrient solution to grow for a further 8 d before commencing the NaCl treatment. Each pot and lid, from which the shoot protruded, was covered with aluminium foil to prevent entry of light. The experiment consisted of two sets (for different samplings) of 24 pots (2 NaCl treatments × 3 stem-infusion treatments × 4 replicates), i.e. 48 pots in total; the treatments and sampling times are described below. The full-strength nutrient solution contained (mM): 5.0 Ca^2+^, 5.0K^+^, 0.625 NH_4_^+^, 0.4 Mg^2+^, 0.2 Na^+^, 5.4 SO_4_^2−^, 4.4 NO_3_^−^, 0.2 H_2_PO_4_^−^, 0.1 SiO_3_^2−^, 0.1 Fe-sequestrene, 0.05 Cl^−^, 0.025 BO_3_^3−^, 0.002 Mn^2+^, 0.002 Zn^2+^, 0.0005 Cu^2+^, 0.0005 MoO_4_^2−^ and 0.001 Ni^2+^. The solution was buffered with 1.0mM MES (2-[N-Morpholino]ethanesulfonic acid) and adjusted to pH 6.5 using KOH.

### Salt treatment application

The 30mM NaCl treatment, the level at which Rupali did not produce mature filled pods (Khan *et al.*, 2015), was applied to half of the pots in two 15mM increments 24h apart when plants were 20 days old. The other half of the pots were maintained as non-saline controls in which the nutrient solution contained 0.2mM Na^+^ and 0.05mM Cl^−^. All nutrient solutions were renewed on a weekly basis and topped up with deionized water as required (initially every second day then daily during the final 4 weeks). Pots were arranged in a completely randomized design and all pots were moved randomly every week to minimize the effects of possible environmental variation within the phytotron. Solution pH was measured every second day and maintained at ~6.5 by additions of KOH as required.

### Stem-infusion treatment application

At the time of flowering (42-day-old plants), plants in each of the NaCl treatments (controls or 30mM NaCl) were either infused with sucrose solution (0.44M; Ψπ=–1.1MPa), infused with water only, or maintained without infusion. The infusion system was modified from Boyle *et al.* (1991) and set up for individual plants. Briefly, the stem was infused using a hypodermic needle (23 gauge; Terumo Corporation, Tokyo, Japan) connected to a flexible plastic tube (inner diameter, 4.0mm; outer diameter, 5.0mm). This plastic tube was connected to a plastic bottle, containing the infusion solution, via an 18 gauge needle inserted into a rubber septum at the base of the bottle. The bottles (one per infused plant) were held in a rack 1.2 m above the pot surface so that the solution would flow slowly into the stems (volumes recorded; see Table 1). The system was examined daily to ensure that there were no leaks or bubbles in the solution continuum. The first infusion was made at the stem base and each week the needle was removed and reinserted at a different place on a primary branch (~2–4cm from the previous insertion; always near the base of these branches); the needle insertion site was moved to ensure no blockages occurred and with the needle briefly removed this also made it easier to renew the nutrient solution and re-randomise pots each week. The amount of infused sucrose was calculated from the total solution intake and its known concentration. The conversion efficiency of sucrose to total plant dry mass (roots+shoots+seeds) was calculated as described in Mooney (1972) where, due to the cost of construction, 100g of sucrose would presumably result in 45.16g of dry matter.

**Table 1.** Sucrose intake and sucrose contribution to dry mass of chickpea (Rupali) subjected to control or 30mM NaCl treatments either without infusion or with stem-infusion with water or 0.44M sucrose (Ψπ=–1.1MPa) Plants were grown in nutrient solution culture and NaCl treatments were imposed on 20-day-old plants for 97 d (20/15±2 °C day/night air temperatures). Infusion treatments were started at the time of flowering (42-day-old plants) and continued until maturity. The data were calculated at maturity and values are means±SE (*n*=4). The sucrose potential contribution to plant dry mass (roots+shoots+seeds) was estimated according to Mooney (1972); see the ‘Materials and methods’. Significant differences (salt × infusion interaction at *P*=0.05) are indicated by different letters for each mean within each column. NA, not applicable: because no data were available in columns of ‘Sucrose intake’ and ‘% of plant dry mass potentially resulting from infused sucrose’ as only water was infused in the water infusion treatment. Plant dry mass (roots+shoots+seeds) in non-infused plants was 109.0±7.1 and 22.4±2.4g in the non-saline and 30mM NaCl conditions, respectively.

Treatments		Total solution intake (ml)	Average daily uptake rate (ml)	Sucrose intake (g dry mass)	Plant dry mass (g)	% of plant dry mass potentially resulting from infused sucrose (%)
Non-saline	Water infusion	157^a^±2	2.1^a^	NA	117.8^a^±4.1	NA
	Sucrose infusion	125^c^±5	1.7^c^	18.8^b^±0.7	104.8^a^±8.8	8.2^b^
30mM NaCl	Water infusion	109^d^±4	1.5^d^	NA	24.3^c^±2.5	NA
	Sucrose infusion	142^b^±5	1.9^b^	21.4^a^±0.7	63.8^b^±3.2	15.2^b^

### Plant samplings for growth

Initial samples were taken at the start of the stem-infusion treatments by sampling one plant from each pot; one set of these ‘initial’ plants (24 plants) were used for growth measurements and the other set (24 plants) was discarded, leaving one plant per pot in each of the two sets of 24 pots for subsequent samplings. There were two subsequent samplings (each from one of the two sets of 24 pots): one at 28 d of infusion treatments and one at 75 d of infusion treatments (at maturity). At 28 d of infusion, tissues were sampled for ion and sugar analyses, and were measured for shoot water potential, leaf sap osmotic potential (described below) and fresh and dry mass. To obtain root samples, the roots were rinsed in 5mM CaSO_4_ for 20s and gently blotted using paper towels to remove external water. The plants were separated into various organs with specific subsamples weighed and used in the various measurements (described below), and remaining tissues also measured for fresh mass and then oven-dried at 65 °C for 48h for dry mass measurements. Similarly, plants at maturity were separated into various organs and then oven-dried at 65 °C for 48h for dry mass measurements. To sample reproductive attributes at maturity, pods were separated from the shoot and the number of mature filled- and empty-pods (pods were individually opened) and seeds counted. Seeds were oven-dried at 40 °C for 48h, after which seed mass was measured.

### Leaf gas-exchange measurements

All measurements were conducted on the second youngest fully-expanded leaf, at 27 d of infusion treatments. Gas-exchange measurements were carried out using an LI-6400XT open gas-exchange system (LI-COR Biosciences Inc., Lincoln, NB, USA) at a photosynthetically active radiation (PAR) of 1500 µmol photons m^−2^ s^−1^ (to measure light-saturated rates; Basu *et al.*, 2007) and a CO_2_ concentration of 380 µmol mol^−1^. The gas-exchange measurements were taken on the same day between 10:00 and 14:00h. The leaf chamber temperature was 20 °C with 60–70% relative humidity. The area of leaf within the chamber was determined by excising the tissues (leaflets) at the end of measurements and using a leaf area scanner (Model LI-3000, LI-COR, LAMBDA Instrument Corporation, USA). LI-6400 software was used to calculate net photosynthetic rate (*A*), stomatal conductance (*g*_s_) and intercellular CO_2_ concentrations (C_*i*_).

### Leaf osmotic potential measurements

At 28 d of infusion treatments, the second youngest fully-expanded leaves (petiole+leaflets) from plants in different treatments were collected between 11:30 and 12:30h into air-tight vials, quickly frozen in liquid N_2_ and kept at –20 °C. Leaf samples were thawed in the vials and then crushed to obtain a sample of tissue sap. The osmotic potential was measured using 10 µl of sap into a calibrated freezing-point depression osmometer (Fiske Associates, Model One-Ten, MA, USA).

### Shoot water potential measurements

Shoot water potential (Ψ_w_) was measured on excised branches (immediately covered with clear Glad Wrap^®^) using a Scholander Pressure Chamber. Samples were taken between 11:30 and 12:30h at 28 d of infusion treatments.

### Total sugars analysis

At 28 d of infusion treatments, tissues were sampled, frozen in liquid N_2_ and freeze-dried so that dry mass and total sugars could be measured. The samples were the second youngest fully-expanded leaf (petiole+leaflets), a ~10cm segment of stem (all leaves removed) at ~20–30cm above the infusion sites, roots and young developing pods (~10–15mm in length with small immature seed/s). Sugars were extracted in 80% ethanol, boiled twice under reflux for 20min and total sugars (hexose equivalents) determined by anthrone (Yemm and Willis, 1954) using a spectrophotometer (Model UV/VIS SP8001, Metertech Inc., Taiwan). Glucose spiked into additional tissue samples was used for recovery check of total sugars, which was 88–90%.

### Tissue ion analysis

Oven-dried samples of different tissues (second youngest fully-expanded leaf, green leaves, stems, roots and seeds) were ground to a fine powder and subsampled for analysis of Na^+^, K^+^ and Cl^−^ following the procedures of Munns *et al.* (2010). In brief, the samples were extracted in 0.5M HNO_3_ by shaking for 48h at room temperature. Diluted samples of the extracts were then analyzed for Na^+^ and K^+^ using a flame photometer (Flame Photometer 410, Sherwood, Cambridge, UK) and, for Cl^−^, a chloridometer (Model 50CL, SLAMED ING. GmbH, Frankfurt, Germany). A reference tissue (broccoli, ASPAC #85), with known ion concentrations, was taken through the same procedures to confirm the reliability of the methods.

### Statistical analysis

Data were subjected to two-way analysis of variance (ANOVA) using Genstat software (VSN International Ltd, Hemel Hempstead, UK) to observe the differences between salt treatments or infusion treatments and to test for a salt × infusion interaction. Means were compared for significant differences using LSD at a significance level of 5%.

## Results

### Effects of salt stress and sucrose infusion on growth

**Fig. 1.**
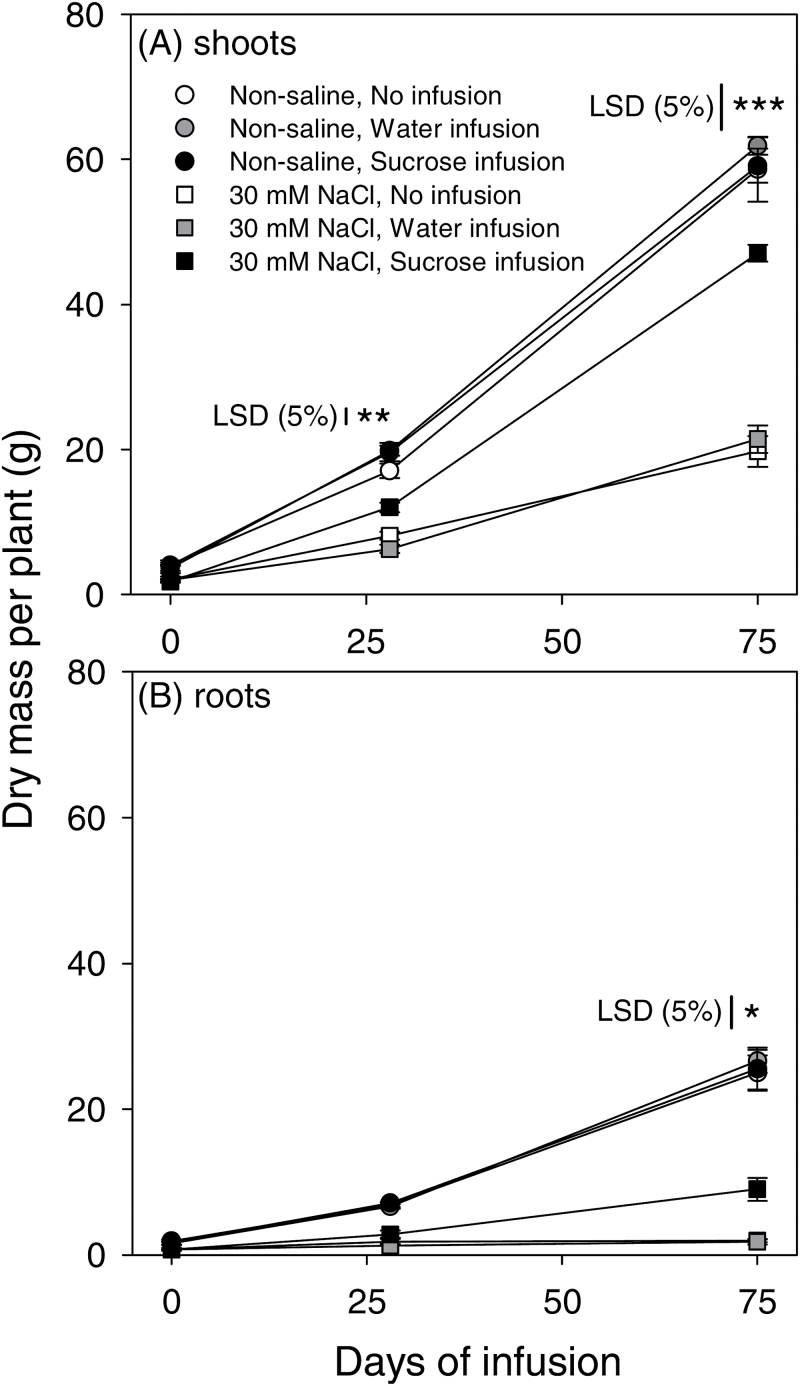
Dry mass of shoots (A) and roots (B) of chickpea (Rupali) subjected to control or 30mM NaCl treatments either without infusion or with stem-infusion with water or 0.44M sucrose (Ψπ=–1.1MPa) and harvested at 0, 28 and 75 d of infusion. Plants were grown in nutrient solution culture and NaCl treatments were imposed on 20-day-old plants for 97 d (20/15±2 °C day/night air temperatures). Infusion treatments were started at the time of flowering (42-day-old plants) and continued until maturity. Symbols represent means±SE (*n*=4). Significant differences among different treatments (salt × infusion interaction at *P*=0.05) are indicated at a specific sampling time (***, significant with *P*<0.001; **, significant with *P*<0.01; *, significant with *P*<0.05) with vertical lines indicating least significant difference (LSD) at *P*=0.05.

The solution intake in all water- and sucrose-infused plants ranged from 1.5–2.1ml d^−1^ (Table 1). The amount of sucrose taken in was 18.8g per plant in the non-saline controls which, if 45.16% was converted to dry matter and the balance respired to provide the energy costs of construction (cf. Mooney, 1972), would be equivalent to ~8.5g tissue dry mass or 8.1% of the total plant dry mass at maturity. In non-saline conditions, the sucrose infusion did not affect shoot or root dry mass of the plants (Fig. 1), presumably since net photosynthesis had declined (see section ‘Effects of salt stress and sucrose infusion on leaf gas-exchange, leaf osmotic potential and shoot water potential’).

The NaCl treatment severely impaired shoot and root growth, but plants with sucrose infusion were less affected than those without the exogenous sucrose provision (Fig. 1). The salt × infusion interaction in the ANOVA was significant for shoot dry mass at 28 and 75 d of infusion (*P*=0.002 and *P*<0.001, respectively) and for root dry mass at 75 d (*P*=0.03). For plants in the NaCl treatment, sucrose infusion increased shoot dry mass by 1.5- and 2.4-fold at 28 and 75 d, respectively, and for roots by 4.6-fold at 75 d, compared with non-infused plants. Plants receiving water infusion did not differ from plants without infusion (Fig. 1). Plants in the NaCl treatment had received 21.4g of sucrose, which with the same assumption above of 45.16% conversion to dry matter (cf. Mooney, 1972), would be equivalent to ~9.7g tissue dry mass or 15.1% of the total plant dry mass at maturity (Table 1). Interestingly, the dry mass of salinized plants with sucrose infusion had increased by 39.5g (Table 1) which was much more than the estimated 9.7g dry mass and even exceeds the 21.4g of the sucrose itself; this higher plant dry mass indicates additional carbon capture by the better maintenance of photosynthetically-active tissues via increased leaf production and branching (see also the next section on reproductive attributes and ‘Discussion’).

The experiment was terminated at 117 d after imbibition; all non-saline plants were alive, whereas only the plants with sucrose infusion were still growing in the NaCl treatment and those with water infusion died at 112±2 d and those without any infusion died at 110±4 d.

### Effects of salt stress and sucrose infusion on reproductive attributes

**Fig. 2.**
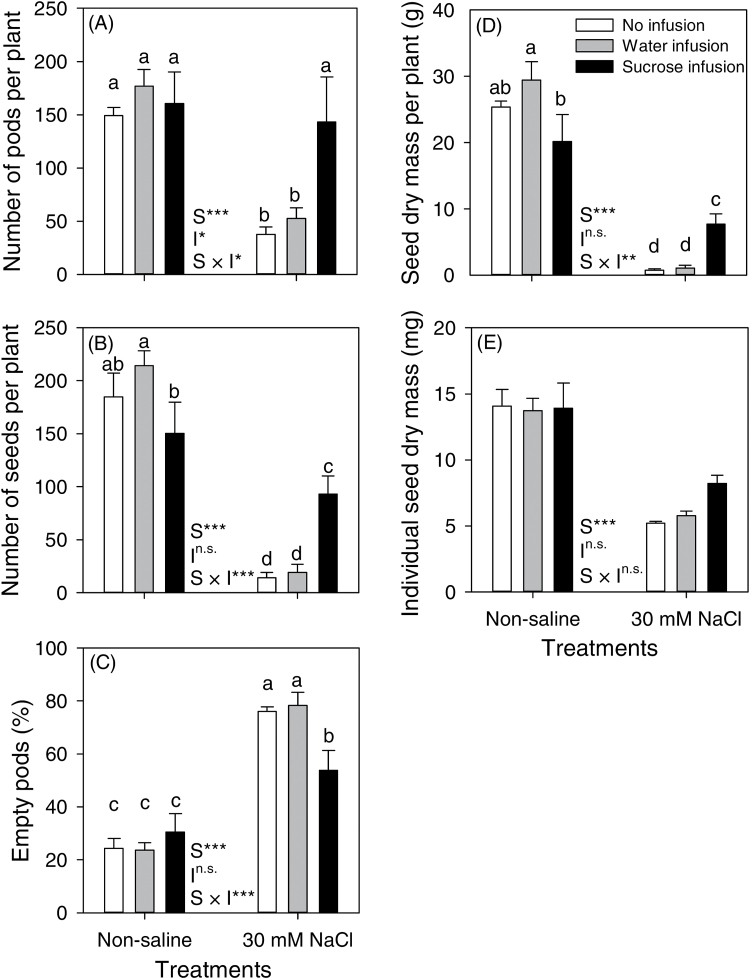
Reproductive attributes of chickpea (Rupali) subjected to control or 30mM NaCl treatments either without infusion or with stem-infusion with water or 0.44M sucrose (Ψπ=–1.1MPa). Plants were grown in nutrient solution culture and NaCl treatments were imposed on 20-day-old plants for 97 d (20/15±2 °C day/night air temperatures). Infusion treatments were started at the time of flowering (42-day-old plants) and continued until maturity. Values are means±SE (*n*=4). Some pods contained two seeds, so seed number is not merely % filled pods × pod number. Two-way ANOVA was used to compare salt (S), infusion (I) and salt × infusion interaction (S × I) effects (***, significant with *P*<0.001; **, significant with *P*<0.01; *, significant with *P*<0.05; ^n.s.^, not significant with *P*>0.05). Significant differences (salt × infusion interaction at *P*=0.05) are indicated by different letters for each mean within each panel.

In non-saline conditions, infusion treatments did not affect the reproductive attributes of plants (Fig. 2). The NaCl treatment had a negative effect on all of the reproductive attributes; however, sucrose infusion improved reproductive performance of the NaCl-treated plants, whereas water-infused plants were similar to the non-infused plants. In the NaCl treatment, sucrose infusion increased the number of pods and seeds per plant by 3.8- and 6.5-fold, respectively, decreased the number of empty pods by 29%, and increased total seed dry mass 10.4-fold, compared with non-infused plants (Fig. 2A–D). The NaCl treatment reduced the individual seed dry mass to 37–42% of the control and salinized plants with sucrose infusion had individual seed dry mass at 58% of control (salt × infusion interaction was not significant, *P*>0.05; Fig. 2E).

### Effects of salt stress and sucrose infusion on total sugars in different tissues

The NaCl treatment reduced the sugars in all tissues and the reduction was greatest in roots (53% of control) and stems (54% of control), followed by leaves (78% of control) and pods (80% of control) of non-infused controls (combined means for non-infused controls and water-infused plants, Table 2). Sucrose infusion increased the sugar concentration in stems (by 46 and 87% in control and NaCl treatment, respectively), leaves (by 44 and 41% in control and NaCl treatment, respectively) and pods (34% in NaCl treatment only), but had no significant effect on the concentration of sugars in the roots (Table 2). The salt × infusion interaction was not significant for total sugars in leaves, stems or roots (*P*>0.1), but it was significant for pods (*P*=0.033) (Table 2).

**Table 2.** Total sugars in developing pods (~10–15mm long with presence of small immature seed/s), second youngest fully-expanded leaves (petiole+leaflets), stems and roots of chickpea (Rupali) subjected to control or 30mM NaCl treatments either without infusion or with stem-infusion with water or 0.44M sucrose (Ψπ=–1.1MPa) Plants were grown in nutrient solution culture and NaCl treatments were imposed on 20-day-old plants for 97 d (20/15±2 °C day/night air temperatures). Infusion treatments were started at the time of flowering (42-day-old plants) and continued until maturity. Tissues were harvested after 28 d of infusion treatments. Values are means±SE (*n*=4). Two-way ANOVA was used to compare salt (S), infusion (I) and salt × infusion interaction (S × I) effects (***, significant with *P*<0.001; **, significant with *P*<0.01; *, significant with *P*<0.05; ^n.s.^, not significant with *P*>0.05). Different upper case superscript letters for means within a column or across a row indicate significant differences between means for salt treatments or infusion treatments at *P*=0.05, respectively, whereas different lower case superscript letters indicate significant differences for salt × infusion interaction at *P*=0.05.

Tissue	Treatment	Total sugars (µmol hexose equivalents g^–1^ EtOH insoluble dry mass)				
		No Infusion	Water Infusion	Sucrose infusion	Mean	Significance
Developing pods	Non-saline	28.9^a^±2.0	28.7^a^±0.7	29.2^a^±1.6	28.9^A^	salt^**^
	30mM NaCl	22.5^b^±1.7	23.5^b^±1.4	30.3^a^±2.0	25.5^B^	infusion^*^
	Means	25.7^B^	26.1^B^	29.7^A^		salt × infusion^*^
Leaves	Non-saline	33.5±2.8	32.9±1.4	48.3±3.4	38.2^A^	salt^***^
	30mM NaCl	26.3±2.8	25.7±1.5	37.1±1.7	29.7^B^	infusion^***^
	Means	29.9^B^	29.3^B^	42.7^A^		salt × infusion^n.s.^
Stems	Non-saline	43.3±1.9	46.6±3.0	63.1±1.5	51.0^A^	salt^***^
	30mM NaCl	22.9±3.1	25.4±1.6	42.9±5.9	30.4^B^	infusion^***^
	Means	33.1^B^	36.0^B^	53.0^A^		salt × infusion^n.s.^
Roots	Non-saline	62.0±7.4	71.3±11.5	75.2±3.6	69.5^A^	salt^***^
	30mM NaCl	37.7±2.4	32.2±0.6	45.7±2.0	38.5^B^	infusion ^n.s.^
	Means	49.9	51.7	60.5		salt × infusion^n.s.^

### Effects of salt stress and sucrose infusion on leaf gas-exchange, leaf osmotic potential and shoot water potential

**Fig. 3.**
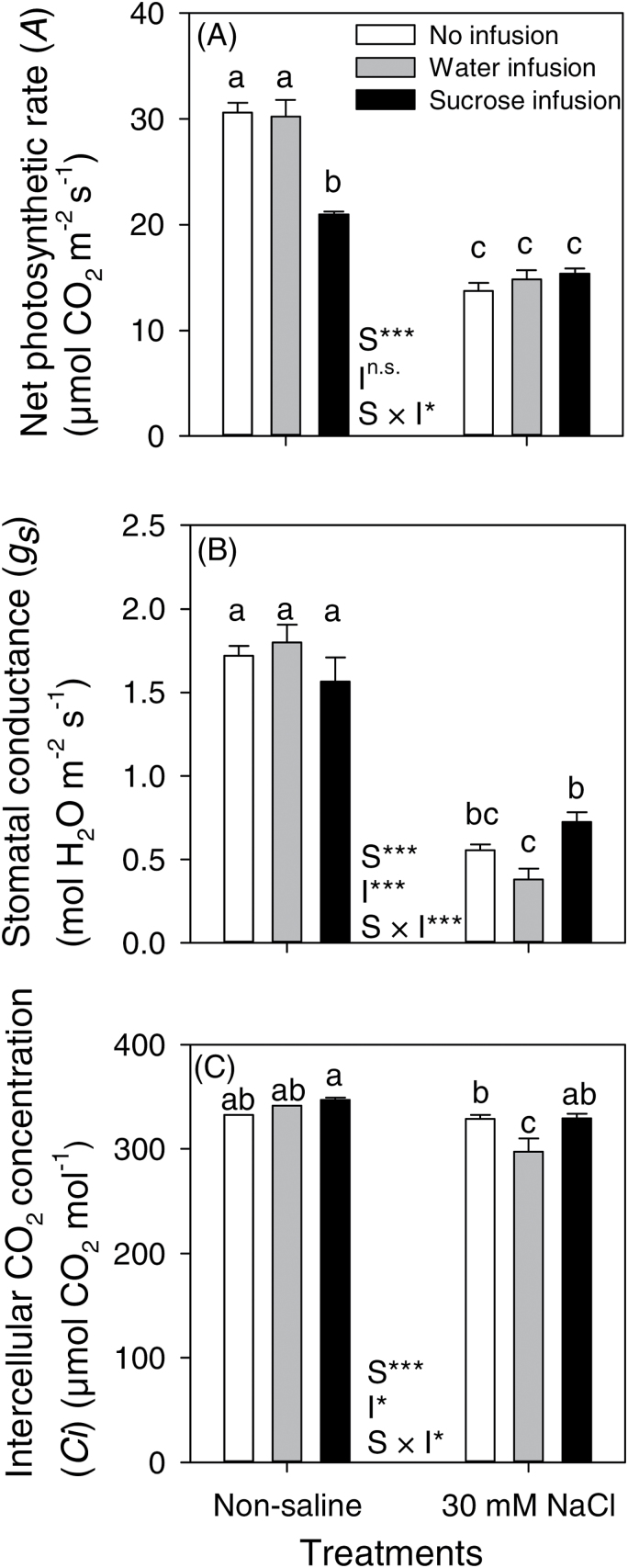
Net photosynthetic rate (A), stomatal conductance (B) and intercellular CO_2_ concentration (C_*i*_) measured on the second youngest fully-expanded leaves of chickpea (Rupali) subjected to control or 30mM NaCl treatments either without infusion or with stem-infusion with water or 0.44M sucrose (Ψπ=–1.1MPa). Plants were grown in nutrient solution culture and NaCl treatments were imposed on 20-day-old plants for 97 d (20/15±2 °C day/night air temperatures). Infusion treatments were started at the time of flowering (42-day-old plants) and continued until maturity. The measurements were taken after 27 d of infusion between 10:00 and 14:00h at a photosynthetically active radiation of 1500 µmol photons m^−2^ s^−1^, a CO_2_ concentration of 380 µmol mol^−1^, leaf chamber temperature of 20 °C and 60–70% relative humidity. Values are means±SE (*n*=4). Two-way ANOVA was used to compare salt (S), infusion (I) and salt × infusion interaction (S × I) effects (***, significant with *P*<0.001; **, significant with *P*<0.01; *, significant with *P*<0.05; ^n.s.^, not significant with *P*>0.05). Significant differences (salt × infusion interaction at *P*=0.05) are indicated by different letters for each mean within each panel.

In non-saline conditions, sucrose infusion inhibited the net photosynthetic rate (*A*) by 31% without affecting stomatal conductance (*g*_*s*_) whereas plants with water infusion and without any infusion behaved similarly for *A* and *g*_*s*_ (Fig. 3A, B). The reduction in *A* in the leaves of plants with sucrose infusion in non-saline conditions was likely caused by feedback inhibition due to the increased level of sugars in leaves (Table 2) (cf. Zhou *et al.*, 1997; Adams III *et al.*, 2013). NaCl treatment reduced *A* and *g*_*s*_ to 54 and 33%, respectively, of the values for non-saline plants (combined means of all infusion treatments). *A* did not differ for plants in the NaCl treatment with the various infusion treatments, although the salt × infusion interaction was significant for *A* (*P*<0.001) owing to the differences among plants with the infusion treatments in the non-saline controls. The NaCl treatment reduced intercellular CO_2_ concentrations (C_*i*_) only by 7% (average of all infusion treatments) and the C_*i*_ was reduced in the salinized plants only when infused with water; however, it did not affect photosynthesis (Fig. 3A, C).

**Fig. 4.**
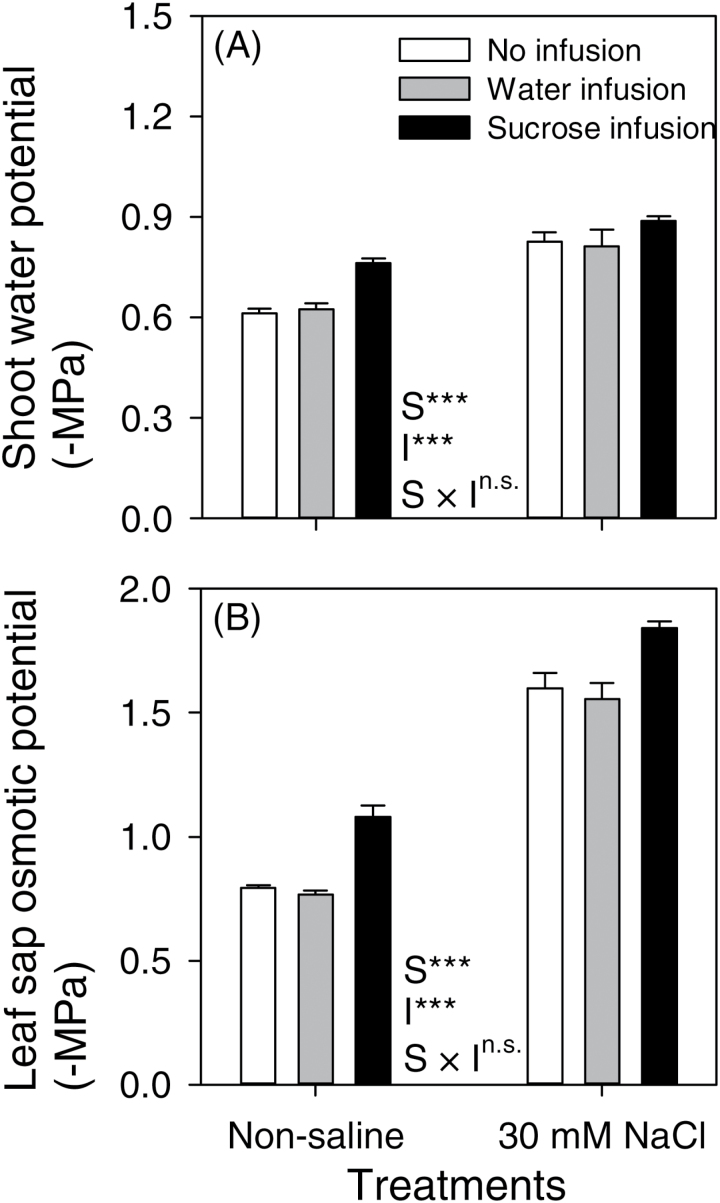
Shoot water potential (A) and leaf sap osmotic potential (B) of chickpea (Rupali) subjected to control or 30mM NaCl treatments either without infusion or with stem-infusion with water or 0.44M sucrose (Ψπ=–1.1MPa). Plants were grown in nutrient solution culture and NaCl treatments were imposed on 20-day-old plants for 97 d (20/15±2 °C day/night air temperatures). Infusion treatments were started at the time of flowering (42-day-old plants) and continued until maturity. The measurements were taken after 28 d of infusion. Leaf sap osmotic potential was measured on the second youngest fully-expanded leaf (petiole+leaflets). Values are means±SE (*n*=4). Two-way ANOVA was used to compare salt (S), infusion (I) and salt × infusion interaction (S × I) effects (***, significant with *P*<0.001; **, significant with *P*<0.01; *, significant with *P*<0.05; and ^n.s.^, not significant with *P*>0.05).

**Fig. 5.**
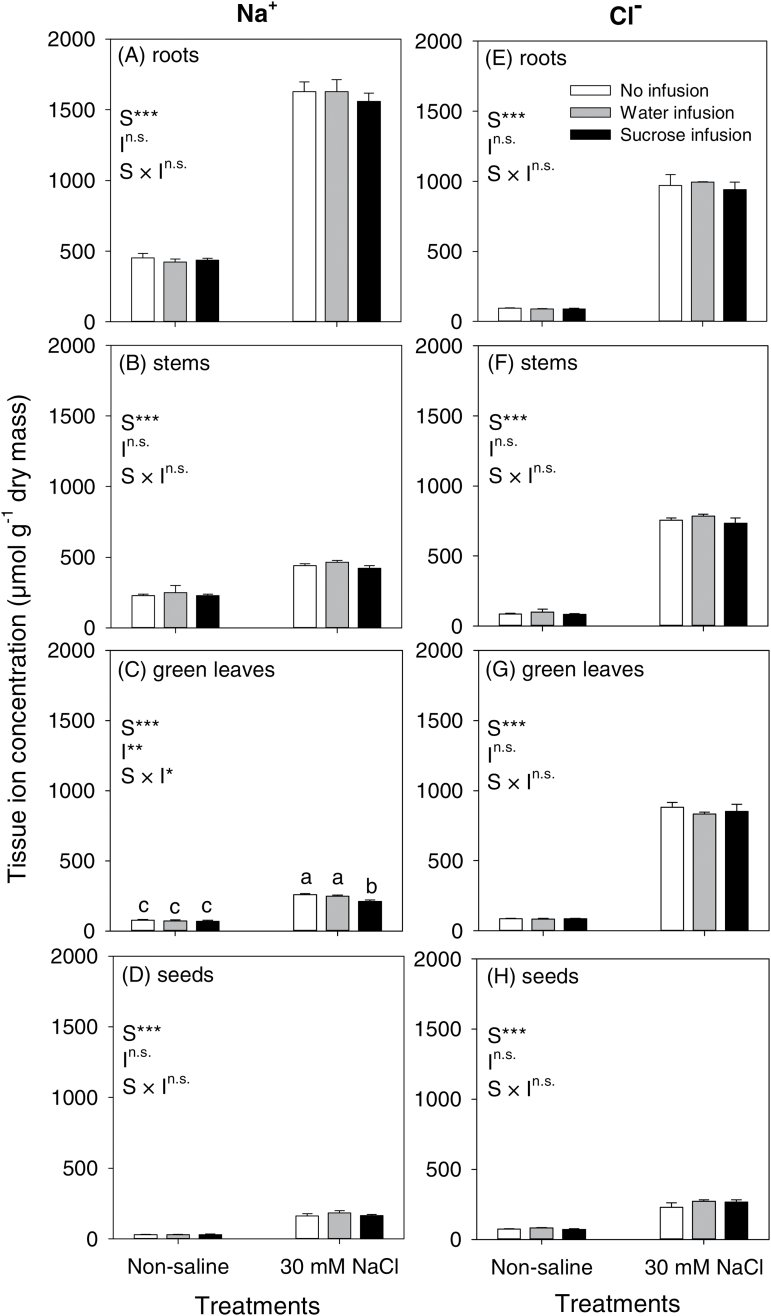
Tissue concentrations of Na^+^ (left column; A–D), and Cl^−^ (right column; E–H) in roots (A, E), stems (B, F), green leaves (C, G) and seeds (D, H) of chickpea (Rupali) subjected to control or 30mM NaCl treatments either without infusion or with stem-infusion with water or 0.44M sucrose (Ψπ=–1.1MPa). Plants were grown in nutrient solution culture and NaCl treatments were imposed on 20-day-old plants for 97 d (20/15±2 °C day/night air temperatures). Infusion treatments were started at the time of flowering (42-day-old plants) and continued until maturity. For measurements of ion concentrations, roots, stems and leaves were harvested after 28 d of infusion whereas seeds were harvested at maturity. Values are means±SE (*n*=4). Two-way ANOVA was used to compare salt (S), infusion (I) and salt × infusion interaction (S × I) effects (***, significant with *P*<0.001; **, significant with *P*<0.01; *, significant with *P*<0.05; ^n.s.^, not significant with *P*>0.05). Significant differences (salt × infusion interaction at *P*=0.05) were only observed for Na^+^ in leaves, which are indicated by different letters for each mean within each panel.

The NaCl treatment lowered both shoot water potential (Ψ_w_) and leaf sap osmotic potential (Ψπ_sap_) (Fig. 4A, B), reflecting the increased concentrations of Na^+^ and Cl^−^ in tissues of plants when exposed to NaCl (Fig. 5; considered in the next subsection). In both the non-saline and NaCl-treated plants, Ψ_w_ and Ψπ_sap_ further decreased in response to sucrose infusion compared to plants with water infusion or no infusion. Water infusion alone did not affect Ψ_w_ or Ψπ_sap_ of the NaCl-treated plants (Fig. 4) and the growth of water-infused plants was similar to plants without infusion under both NaCl treatments (Fig. 1). Additionally, the volume of infusion solution per day was low (~2ml d^−1^) compared with the total water flux per day through the sucrose-infused salinized plants (~63ml d^−1^) measured 14–15 d after infusion commenced (data not shown). Thus, the infusion treatment of water alone tested the possible impacts of this small additional water supply and water-infused plants did not differ from non-infused plants in any aspect. Leaf Ψπ_sap_ decreased due to increased concentrations of ions (Na^+^ and Cl^−^) and total sugars in leaves (Table 2, Figs 4, 5). However, sugars contributed only 0.9–2.2%, compared with Na^+^, K^+^ and Cl^−^ which altogether contributed 35–65%, towards Ψπ_sap_ of plants in the different treatments (Table 3). Moreover, the change in Ψπ_sap_ in response to the NaCl treatment (97–103% increases in non- and water-infused plants) was more than the change due to sucrose infusion (29–37% in control and NaCl-treated plants) (Supplementary Table S1 at *JXB* online).

**Table 3.** Leaf tissue water content, leaf sap osmotic potential (Ψπ_sap_) and estimation of contribution of ions and sugars towards leaf Ψπ_sap_ of chickpea (Rupali) subjected to control or 30mM NaCl treatment either without infusion or with stem-infusion with water or 0.44M sucrose (Ψπ=–1.1MPa) Plants were grown in nutrient solution culture and NaCl treatment was imposed on 20-day-old plants for 97 d (20/15±2 °C day/night air temperatures). Infusion treatments were started at the time of flowering (42-day-old plants) and continued until maturity. Measurements were taken on the second youngest fully-expanded leaf (petiole+leaflets) after 28 d of infusion treatments, individually for each measurement using samples from different branches of the same plant within each replicate. Values are means±SE (*n*=4; i.e. four plants in four different pots per treatment). Leaf Ψπ_sap_ data are taken from Fig. 4 and the tissue water content data below and tissue ion (Fig. 5; Supplementary Fig. S1) and sugar data (Table 2) were used for the calculation of estimated contributions to leaf Ψπ_sap_. The contribution of a given solute was calculated as: Ψπ=−nRT/V, where n is the number of solute molecules; R, the universal gas constant; T, temperature in °K; and V, volume in L. Values are means±SE (*n*=4). The value for contribution of sugars would be an overestimate as it uses the total hexose units from Table 2, of which a proportion would have been present in the tissues as sucrose. The osmotic coefficient of the NaCl in the external solution and of Na^+^ and Cl^−^ in the tissues was assumed to be 1.

Treatments		External Ψπ (MPa)	Leaf Ψπ_sap_ (MPa)	Leaf water content (ml g^–1^ dry mass)	Estimated contributions of ions and sugars to leaf Ψπ_sap_ (%)				
					Na^+^	Cl^−^	K^+^	Total ions	Sugars
Non-saline	No infusion	−0.04	−0.79±0.01	5.3±0.2	4.3±0.4	5.3±0.2	36.0±0.8	45.7±1.1	2.0±0.0
	Water infusion	−0.04	−0.77±0.02	5.2±0.2	4.2±0.1	5.3±0.4	37.3±2.0	46.8±1.9	2.0±0.1
	Sucrose infusion	−0.04	−1.08±0.05	5.1±0.3	3.1±0.2	4.0±0.5	28.1±2.3	35.2±2.7	2.2±0.1
30mM NaCl	No infusion	−0.16	−1.60±0.06	4.6±0.2	8.5±0.4	24.6±5.7	23.3±1.3	56.4±6.6	0.9±0.1
	Water infusion	−0.16	−1.56±0.07	4.4±0.1	9.6±0.4	29.5±2.1	26.5±0.8	65.5±3.0	0.9±0.1
	Sucrose infusion	−0.16	−1.84±0.03	4.8±0.1	7.5±0.3	22.6±1.1	19.6±1.2	49.7±2.4	1.0±0.1

### Effects of salt stress and sucrose infusion on Na^+^, Cl^−^ and K^+^ concentrations in different tissues

In non-saline conditions, with the various infusion treatments, plants had similar Na^+^ concentrations in each of the tissues (roots, stems and leaves). Na^+^ concentration increased in all tissues of plants in the NaCl treatment but was 15–18% lower in leaves of sucrose-infused plants than those of plants without infusion or with only water infusion (Fig. 5C). Thus, the NaCl × infusion interaction for Na^+^ concentration was significant for leaves (*P*=0.0146) but not for roots (*P*=0.67) or stems (*P*=0.86) (Fig. 5A–C). The Cl^−^ concentrations increased in all tissues of plants in the NaCl treatment (Fig. 5) but unlike the situation for leaf Na^+^ concentration, the sucrose infusion treatment did not influence leaf Cl^−^ concentration (Fig. 5G). Similarly, K^+^ concentrations in the roots, stems and leaves were similar within each tissue type for plants in the various NaCl and infusion treatments (no significant NaCl × infusion interaction for K^+^ concentration in roots (*P*=0.26), stems (*P*=0.80) or leaves (*P*=0.37) (Supplementary Fig. S1).

In seeds, the tissue Na^+^ and Cl^−^ concentrations were both low in non-saline conditions but increased 5.6- and 4.1-fold in the NaCl treatment. The stem-infusion treatments did not influence the Na^+^ and Cl^−^ concentration in the seeds (no significant salt × infusion interaction, *P*=0.44 and *P*=0.30 for Na^+^ and Cl^−^, respectively; Fig. 5D, H). Seeds contained K^+^ at a much higher concentration than Na^+^ (particularly in non-saline conditions), and the NaCl treatment increased the K^+^ concentration in seeds of plants without any infusion treatment, but had no effect on seed K^+^ of plants with either water only or sucrose infusion (significant NaCl × infusion interaction, *P*=0.005; Supplementary Fig. S1).

## Discussion

We tested the hypothesis that limited assimilate supply contributes to reproductive failure in salt-stressed chickpea, by supplying exogenous sucrose via a stem-infusion technique. The 30mM NaCl treatment impeded photosynthesis and tissue sugar concentrations declined, but sucrose infusion resulted in a 10.4-fold increase in seed yield per plant. In contrast, water infusion as a negative control did not benefit the salinized plants and sucrose infusion did not enhance non-saline control plants. The partial rescue of reproduction by increased sucrose supply demonstrates that low availability of assimilates is one factor hindering reproductive growth in salt-stressed chickpea.

Sucrose infusion into stems has indicated the importance of assimilate supply for reproductive growth in water-stressed maize (Boyle *et al.*, 1991; Zinselmeier *et al.*, 1995a, 1999) and soybean (Abdin *et al.*, 1998; Zhou *et al.*, 2000). Our study is the first report to use stem-infusion of sucrose to test the hypothesis that low assimilate availability limits reproductive growth in a salt-sensitive crop species. Salinity decreased the availability of assimilates in chickpea (Table 2) by reducing photosynthesis per unit leaf area (Figs 1, 3) and shoot size (Fig. 1; leaf area was not measured but would, like dry mass, be less), but sucrose infusion increased both the vegetative and the reproductive growth (numbers of pods and seeds, and, importantly, seed yield). Continued vegetative growth produces reproductive sites and so better maintenance of above-ground biomass is positively related to seed numbers in salt-stressed chickpea (Pushpavalli *et al.*, 2015). Our finding of insufficient assimilates for reproductive growth can explain earlier observations of reproductive failure in salt-stressed chickpea (Vadez et al., 2007, 2012; Samineni *et al.*, 2011; Kotula *et al.*, 2015).

In addition to assimilate limitation, reproductive development in some salt-stressed plants can be affected by high concentrations of Na^+^ and Cl^−^ in reproductive tissues (e.g. rice, Khatun and Flowers, 1995; Khatun *et al.*, 1995), but for chickpea we consider that ion toxicity in the reproductive tissues was unlikely. Salinized chickpea infused with sucrose attained similar pod numbers as plants in non-saline conditions (Fig. 2), indicating that provided the assimilate supply is adequate, fertilization and pod retention can proceed in saline conditions. Additionally, recent studies of salt-stressed chickpea have reported low levels of Na^+^ in reproductive tissues that are unlikely to adversely affect reproductive processes (Kotula *et al.*, 2015; Pushpavalli *et al.*, 2016) and pollen viability, *in vitro* pollen germination or *in vivo* pollen-tube growth in flowers were not impacted (Turner *et al.*, 2013). Similarly, reproductive development in wheat and barley was independent of Na^+^ and Cl^−^ accumulation in the apex, which was considered too low to affect metabolism (Munns and Rawson, 1999). In the present study, seed Na^+^ and Cl^−^ concentrations did not differ for salinized plants with the various stem-infusion treatments and K^+^ concentration was slightly lower in seeds of sucrose-infused plants (Fig. 5, Supplementary Fig. S1). Thus, considering the results described above for chickpea, the beneficial effect on seed production from the supply of exogenous sucrose highlights that assimilates limit reproductive success in salt-stressed chickpea, rather than a direct ion toxicity in reproductive tissues.

Sucrose infusion increased seed yield via increased pod numbers and more filled pods in a salt-sensitive genotype (Rupali) of chickpea (Fig. 2), a response consistent with salt tolerance in chickpea being associated with the production of more flowers and pods and with a higher proportion of filled pods in salt-tolerant than salt-sensitive genotypes (Vadez *et al.*, 2012; Kotula *et al.*, 2015). In maize subjected to drought, sucrose infusion increased grain number and grain weight by rescuing embryo abortion and improving embryo growth (Boyle *et al.*, 1991; Zinselmeier *et al.*, 1995a). However, the rescue of reproductive growth by exogenous sucrose for salinized chickpea was only partial; although pod numbers were similar to non-salinized controls, when infused with sucrose the salt-stressed plants had a 2.2-fold higher percentage of empty pods, ~50% fewer seeds and ~42% lower dry mass per seed, compared with non-saline plants without infusion (Fig. 2). So, while sucrose infusion decreased pod and seed abortion, it did not completely alleviate the deleterious effects of salt stress on pod filling (i.e. seed growth). Seed growth is primarily supplied with assimilates from current photosynthesis in leaves (Singh and Pandey, 1980; Fader and Koller, 1985; Westgate and Boyer, 1985; Khan and Abdullah, 2003; Turner *et al.*, 2005) and photosynthates produced after anthesis provided up to 98% of the seed carbon in chickpea (Turner *et al.*, 2005). The prevalence of salt-induced seed abortion, even in sucrose-infused chickpea, is perhaps still linked with a suboptimal availability of assimilates owing to reduced shoot growth (Fig. 1; i.e. less photosynthetic tissue) and reduced rates of photosynthesis (Fig. 3) so that whole-plant carbon gain was still lower in these salinized plants than in controls. Future work could attempt to infuse greater volumes or higher concentrations of sucrose solution. Reproductive failure in chickpea under salt stress may also be related to hormonal changes in salinized plants (Albacete *et al.*, 2014), but hormone physiology was beyond the scope of the present experiment.

Salt stress reduced the concentration of sugars in the various organs of chickpea, but sucrose infusion via the stem of plants in the NaCl treatment increased the total sugars in leaves, stems and pods, but not in the roots (Table 2). The 34% increase in the concentration of total sugars in pods of the NaCl-treated plants with sucrose infusion could have resulted in the increased growth of seeds, although growth rates of seeds can be determined both by tissue sucrose concentration and by the flux of sucrose into the growing seed; i.e. if sucrose delivery to the pod and uptake by seeds for growth both change in concert, tissue concentrations can remain relatively stable (e.g. soybean, Fader and Koller, 1985). In drought-stressed maize with stem-infused sucrose, the stems and leaves were the primary sinks for the infused sucrose (60% of the infused sucrose) whereas only small amounts (6% of the infused sucrose) accumulated in the reproductive tissues (Zinselmeier *et al.*, 1995a), nevertheless reproductive growth was enhanced (Zinselmeier et al., 1995a, b). Chickpea is an indeterminate species and, during reproductive development, young vegetative tissues may directly compete with developing seeds for assimilate supply (Turner *et al.*, 2005). Chickpea apparently invested the infused sucrose into vegetative growth (which would provide leaf area for photosynthesis) and the increased branch numbers provided more reproductive sites, but the pods perhaps did not receive sufficient assimilates for seed growth due to reduced photosynthesis in the leaves of salt-stressed plants (Fig. 3).

Water deficits can be a major component of salt stress in plants (Munns, 2002), depending on the salt concentration and thus the osmotic potential. The ability of chickpea to osmotically adjust to soil water deficits has been documented (Leport *et al.*, 1998; Turner *et al.*, 2007; Fang *et al.*, 2010). In the present study, chickpea showed greater declines in leaf sap Ψπ than the decline of −0.16MPa for 30mM NaCl (Table 3). The contribution to leaf sap Ψπ of sugars was only ~1–2%, whereas K^+^, Na^+^ and Cl^−^ made larger contributions (Fig. 4, Table 3, Supplementary Table S1). The Ψπ of 30mM NaCl (−0.16MPa) is relatively small and when this Ψπ was imposed on chickpea in a concentrated macronutrient culture solution for 42 d, the leaves showed osmotic adjustment and there was no impact on vegetative growth (Khan *et al.*, 2016). Thus, the improved performance of sucrose-infused chickpea in 30mM NaCl was not associated with additional osmotic adjustment in leaves, but rather (as discussed above) the sucrose infused into the stem added to the limited assimilate availability in the salt-damaged plants and promoted vegetative and reproductive growth.

## Conclusions

By infusion of sucrose into the stem, this study demonstrated that low assimilate supply contributes to reproductive failure in salt-stressed chickpea. Salt stress reduced reproductive success in chickpea via the reduced availability of assimilates, caused by reduced photosynthesis and less shoot growth which lowered leaf area as well as branching and, therefore, flower and pod numbers, and reduced seed growth. Sucrose infused into the stem increased total sugars in different tissues and partially alleviated the NaCl-induced reproductive failure. Inadequate assimilate supply is one cause of reproductive failure in chickpea under salt stress and other possible factors should be explored within this context.

## Supplementary data

Supplementary data are available at *JXB* online.

Figure S1. Tissue concentrations of K^+^ in roots, stems, green leaves and seeds of chickpea (Rupali) subjected to control or 30mM NaCl treatments either without infusion or with stem-infusion with water or 0.44M sucrose (Ψπ=–1.1MPa).

Table S1. Salt stress and sucrose infusion induced changes in the leaf sap osmotic potential of chickpea (Rupali) subjected to control or 30mM NaCl treatments either without infusion or with stem-infusion with water or 0.44M sucrose (Ψπ=–1.1MPa).

## Supplementary Material

supplementary_figure_S1_table_S1
