# Supplementary material for: Vegetative and reproductive growth of salt-stressed chickpea are carbon-limited: sucrose infusion at the reproductive stage improves salt tolerance
Source: J Exp Bot. 2016 May 2;68(8):2001–11. doi: 10.1093/jxb/erw177 (PMC5429013; doi:10.1093/jxb/erw177)
Supplement: supplementary_figure_S1_table_S1 [file erw177_suppl_supplementary_figure_S1_table_S1.pdf]

Journal of Experimental Botany

Vegetative and reproductive growth of salt-stressed chickpea are carbon-limited: sucrose infusion at the reproductive stage improves salt tolerance.

Hammad A. Khan, Kadambot H. M. Siddique and Timothy D. Colmer

SUPPLEMENTARY DATA

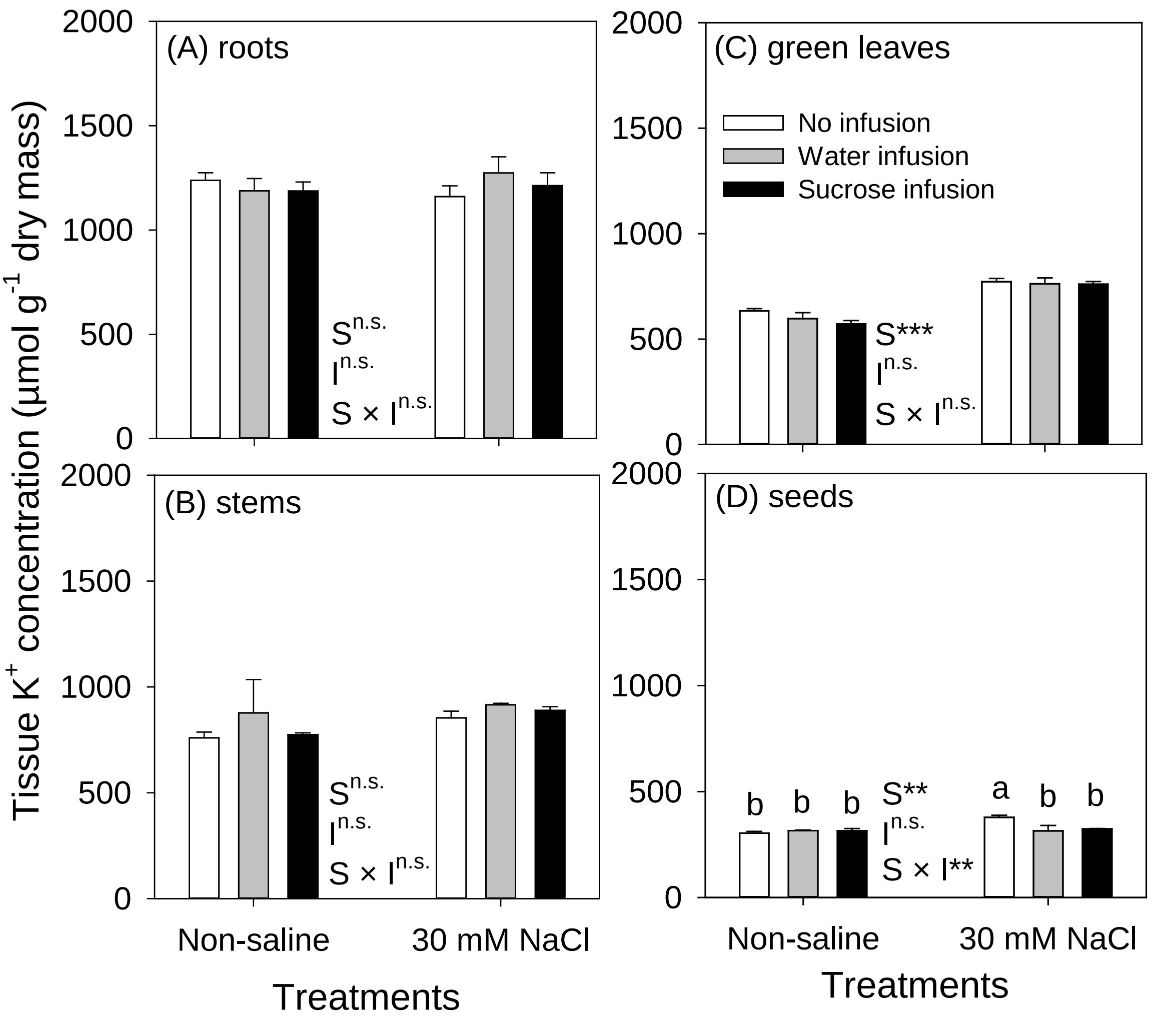

Fig. S1. Tissue concentrations of K^+ in roots (A), stems (B), green leaves (C) and seeds (D) of chickpea (Rupali) subjected to control or 30 mM NaCl treatments either without infusion or with stem-infusion with water or 0.44 M sucrose ($\Psi\pi = -1.1$ MPa). Plants were grown in nutrient solution culture and NaCl treatments were imposed on 20-day-old plants for 97 days (20/15 ± 2 °C day/night air temperatures). Infusion treatments were started at the time of flowering (42-day-old plants) and continued until maturity. For measurements of ion concentrations, roots, stems and leaves were harvested after 28 days of infusion whereas seeds were harvested at maturity. Values are means ± SE (*n* = 4). Two-way ANOVA was used to compare salt (S), infusion (I) and salt × infusion (S × I) effects (*** significant with $P < 0.001$, ** significant with $P < 0.01$, * significant with $P < 0.05$, and $^{n.s.}$ not significant with $P > 0.05$). Significant differences (salt × infusion interaction at $P = 0.05$) were only observed for K^+ in seeds which are indicated by different letters for each mean within each panel.

Table S1. Salt stress and sucrose infusion induced change in the leaf sap osmotic potential of chickpea (Rupali) subjected to control or 30 mM NaCl treatments either without infusion or with stem-infusion with water or 0.44 M sucrose ($\Psi\pi$ = −1.1 MPa). Plants were grown in nutrient solution culture and NaCl treatments were imposed on 20-day-old plants for 97 days (20/15 ± 2 °C day/night air temperatures). Infusion treatments were started at the time of flowering (42-day-old plants) and continued until maturity. Leaf sap osmotic potential ($\Psi\pi_{sap}$) was measured on the 2nd youngest fully-expanded leaf (petiole + leaflets) of plants after 28 days of stem infusion. Values are means ± SE (*n* = 4).

Treatments		Treatment solution $\Psi\pi$ (MPa)	Leaf $\Psi\pi_{sap}$ (MPa)	Salt stress induced change in leaf $\Psi\pi_{sap}$ (MPa)	Sucrose infusion induced change in leaf $\Psi\pi_{sap}$ (MPa)
Control	No infusion	–0.04	–0.79 ± 0.01	-	-
	Water infusion	–0.04	–0.77 ± 0.02	-	-
	Sucrose infusion	–0.04	–1.08 ± 0.05	-	–0.29
30 mM NaCl	No infusion	–0.16	–1.60 ± 0.06	–0.81	
	Water infusion	–0.16	–1.56 ± 0.07	–0.77	
	Sucrose infusion	–0.16	–1.84 ± 0.03	-	–0.23
